# Measuring herpes zoster disease burden in São Paulo, Brazil: a clinico-epidemiological single-center study

**DOI:** 10.6061/clinics/2018/e243

**Published:** 2018-07-13

**Authors:** João Toniolo-Neto, Eliofotisti Psaradellis, Angela Karellis, Emmanouil Rampakakis, Talita Y. Rockett, John S. Sampalis, Kelly D. Johnson, Homero A. Monsanto, Camilo J. Acosta

**Affiliations:** I Nucleo de Pesquisas Clinicas e Envelhecimento (NUPEQ), Universidade Federal de Sao Paulo – Escola Paulista de Medicina (Unifesp-EPM), Sao Paulo, SP, BR; II JSS Medical Research Inc., St-Laurent, QC, Canada; III Center for Observational & Real-World Evidence, Vaccines, Merck & Co., Inc., Kenilworth, NJ, United States; IV Center for Observational & Real-World Evidence, Latin America, MSD (IA) LLC, Carolina, Puerto Rico

**Keywords:** Herpes Zoster, Observational Study, Brazil, Pain

## Abstract

**OBJECTIVES:**

Herpes zoster is characterized by acute neuritis and post-herpetic neuralgia. Currently, data concerning the zoster-associated impact on quality of life and healthcare resource utilization in Brazil are scarce. This study measured the zoster-associated burden in a Brazilian population.

**METHODS:**

This was a prospective, observational, single-cohort study conducted in a primary hospital’s emergency room in São Paulo, Brazil. Patients enrolled at various timepoints during a zoster episode were followed over 180 days. The Zoster Brief Pain Inventory and the Initial Zoster Impact Questionnaire assessed zoster-associated pain. The EuroQoL assessed the impact of herpes zoster and/or zoster-associated pain on quality of life. Healthcare resource utilization was assessed by patient-reported questionnaires.

**RESULTS:**

One-hundred forty-six zoster patients were enrolled [mean (SD) age of 69.9 (10.9) years]. Mean (SD) worst pain scores decreased from 5.3 (3.5) at baseline to 1.9 (3.0) 180 days following rash onset. Mean (SD) EuroQoL scores significantly decreased from 0.9 (0.2) before rash appearance to 0.7 (0.2) after rash onset (*p*<0.001), followed by gradual improvements in quality of life over 180 days, with pre-herpes zoster quality of life achieved at the end of the observation period. The majority of patients purchased prescription medications (89.7%) and required doctor’s office visits (65.8%) for zoster episodes.

**CONCLUSIONS:**

Herpes zoster is associated with a significant disease burden, including zoster-associated pain, impaired quality of life and increased healthcare resource utilization in Brazil. These results support the implementation of early intervention and prevention programs such as vaccinations to reduce the herpes zoster-associated disease burden in Brazil.

## INTRODUCTION

Herpes zoster (HZ), also known as shingles or zoster, is caused by the reactivation of the varicella-zoster virus (VZV) in sensory ganglia after a latency period following an initial infection with varicella 1.

The most significant features associated with HZ are a blistering dermatomal rash, pain, acute neuritis and, later, post-herpetic neuralgia (PHN) 2. PHN has been known to cause disordered sleep, chronic fatigue, anxiety and severe depression. Studies have shown that HZ can severely affect elderly patients’ functional capacity and quality of life (QoL) at levels comparable to the effects of incapacitating comorbidities such as cardiovascular disease, diabetes and respiratory illnesses 3,4.

PHN is characterized by tender, burning, throbbing, stabbing, shooting and/or sharp pain persisting for months or years, which may lead to emotional distress. The manifestations may be constant or paroxysmal as minor stimuli to the affected skin may trigger allodynia. At least 90% of patients with PHN experience allodynia, and it is generally one of the most distressing and debilitating type of pain 5. Pain has been shown to persist for more than 3 months and for more than one year in 30-50% and 20% or more, respectively, of elderly patients with PHN 6.

In the United States, the incidence of HZ has been reported to be 10-20% in the general population 7 and 60-70% among individuals older than 50 years of age 8.

Currently, published data are limited concerning the clinical characteristics, disease severity and health care resource utilization (HCRU) related to HZ in Brazil. Given the aging Brazilian population, the HZ disease burden will increase in the near future. A thorough understanding of the Brazil-specific HZ burden and associated HCRU would help assess the potential impact of a HZ vaccination program in Brazil. The aim of this study was to measure the HZ disease burden, assess the impact on QoL and determine the HCRU due to HZ episodes in a Brazilian population.

## MATERIALS AND METHODS

### Study design

The present study is part of the international MASTER (Monitoring and Assessing Shingles Through Education and Research) study conducted in 8 countries (Canada, Brazil, Costa Rica, Mexico, Argentina, Taiwan, South Korea and Thailand) aimed to assess the disease burden associated with HZ and PHN 12-17. Within Brazil, this was an observational, prospective cohort and prevalence study of patients presenting with zoster-associated pain (ZAP), including acute pain during HZ episodes and PHN in an emergency room in São Paulo, assessed by one geriatrician. As patients were recruited at various stages of their zoster episode and were followed for 6 months thereafter, the cohort was considered both prevalent and prospective.

Review board approval was obtained in compliance with local law. The study was conducted in accordance with the International Conference on Harmonization Guidelines for Good Clinical Practices and the tenets of the Declaration of Helsinki. Eligible patients who provided written informed consent prior to cohort entry were included in the study.

### Study population

Patients were prospectively recruited between May 2008 and October 2009 and entered in the cohort at the time of presentation with HZ rash or ZAP symptoms.

Up to 120 patients from a public primary hospital’s emergency room (ER) were planned to be recruited in the study. Patients were part of the nation’s Unified Health System, the ‘Sistema Único de Saúde’ (SUS). Incident cases were defined as patients recruited for a current HZ episode with a duration of less than 7 days. Prevalent cases were defined as subjects enrolled while visiting an investigator for a current HZ episode with a duration of more than 7 days and for which the rash onset had been recorded in the patient’s medical records. Disease onset was defined according to the HZ rash onset date.

Patients were required to be at minimum of 50 years of age at the HZ rash onset date and to have a diagnosis of HZ rash or ZAP (acute pain or PHN) confirmed by a physician. Patients were further expected to have telephone contact and be capable of completing the patient questionnaires, understanding the trial and the content of the informed consent, participating in the study by signing the consent form and being available for the follow-up period of the study.

### Study schedule

Following the baseline assessment (day 0) conducted at the ER, nine additional assessments were performed on days 7, 14, 21, 30, 60, 90, 120, 150 and 180 following study enrollment. The follow-up period lasted six months, and the same schedule was followed for all patients regardless of their disease stage at enrollment. Data were obtained by telephone contact.

### Outcome measures

Burden of disease due to HZ pain was assessed as a function of pain duration, and severity was measured by the Zoster Brief Pain Inventory (ZBPI) and by the Initial Zoster Impact Questionnaire (IZIQ) 18. The ZBPI and IZIQ are zoster-specific questionnaires based on the Brief Pain Inventory (BPI), the latter of which has been validated in a Brazilian population 18,19. PHN was defined as the worst pain in the last 24 hours being scored at least 3 on a 0-10 scale, where a score of 0 represents no pain and a score of 10 represents the worst possible pain, after a minimum of 90 days since rash onset. The EuroQoL (EQ-5D) assessed the impact of HZ and/or ZAP on QoL, with higher index scores indicating a better QoL 20. Patients completed the EQ-5D questionnaire twice, once at baseline, in order to capture the initial QoL, ZAP or PHN, and again during their HZ episode. The EQ-5D questionnaire was further administered at each follow-up assessment date. Latin America weight coefficients were used to provide population-specific preferences for the health states defined by the EQ-5D 20.

Patient-reported HCRU was assessed with the aid of a simple questionnaire administered at each visit. Patients provided information about the health care services utilized due to HZ rash or ZAP since their last visit. HCRU included visits to the ER and admission to a hospital, as well as the use of other health-related services, including use of an ambulance or nursing service, visits to a doctor’s office, physiotherapist/rehabilitation, psychiatrist/psychologist/counselor and visits to a specialist.

Out-of-pocket expenses for health care, including fees for prescription, over-the-counter (OTC) and alternative medications were recorded in a healthcare cost questionnaire. The Work and Productivity questionnaire measured indirect costs, including the number of missed work days by patients or caregivers, duration of extended sick leave, and duration of disability from HZ or ZAP episodes since the last visit.

### Statistical analysis

Descriptive statistics were produced for all variables studied. For continuous variables, measures of central tendency (mean) and dispersion (standard deviation (SD)) were reported, and frequency distributions were produced for all categorical variables. A paired-samples Student’s t-test was used to assess the change in QoL from baseline following HZ rash appearance. The level of statistical significance was set to 0.05. All statistical analyses were performed using the Statistical Analysis Software (SAS Institute, Cary, NC, USA).

## RESULTS

A total of 146 eligible patients were enrolled in the study. During the follow-up period, 139, 137, 138, 142, 146, 144, 143, 145 and 144 patients completed visits at days 7, 14, 21, 30, 60, 90, 120, 150 and 180, respectively.

Table 1 presents the socio-demographic and baseline disease characteristics of the cohort. The mean (SD) age was 69.9 (10.9) years old. The majority of patients were female (64.4%), Caucasian (78.8%) and retired (47.3%). The most common highest level of education was primary/elementary school (47.3%) (Table 1A).

At baseline, 78.1% of patients presented with a HZ rash. The most common affected primary dermatome was the thoracic region (38.4%), followed by the cervical (15.1%) and lumbar (13.7%) regions. Nearly half of patients (48.6%) presented with 1 to 20 lesions in the primary and adjacent dermatomes (Table 1B).

Prior to HZ rash onset, 54.1% of patients experienced ZAP, with mean (SD) worst and average IZIQ scores of 7.4 (2.5) and 5.8 (2.5), respectively. Following HZ rash onset, 91.1% of patients reported ZAP, with mean (SD) worst and average IZIQ scores of 8.6 (7.2) and 5.5 (2.5), respectively. Approximately half of patients (49.3%) experienced PHN. Among the 140 (95.9%) patients who received HZ treatment, the most frequently utilized medication types included analgesics (34.2%), antivirals (27.4%), topical treatments (21.2%) and other therapies (44.5%) (Table 1B).

**Figure 1:**
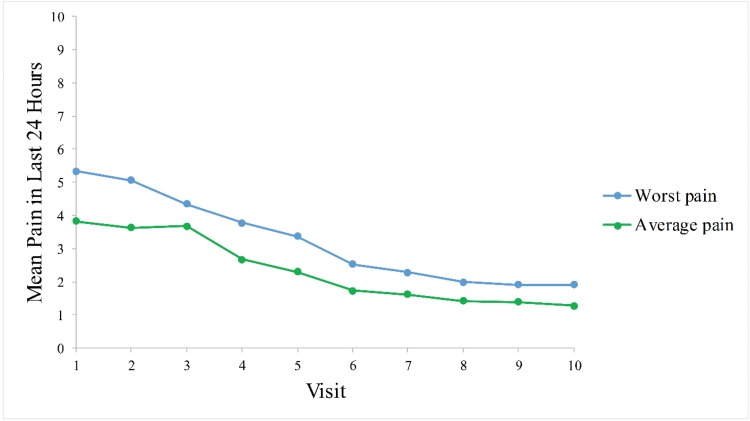
Mean Herpes-Zoster-Associated Worst Pain and Average Pain in the Last 24 Hours over Time. Higher ZBPI scores indicate worse pain.

Figure 1 presents data for ZAP experienced in the last 24 hours over time. The mean (SD) worst ZBPI scores decreased from 5.3 (3.5) at baseline to 1.9 (3.0) at day 180. Similarly, the mean (SD) average ZBPI scores decreased from 3.8 (2.8) to 1.3 (2.2) over the 180-day period.

**Figure 2:**
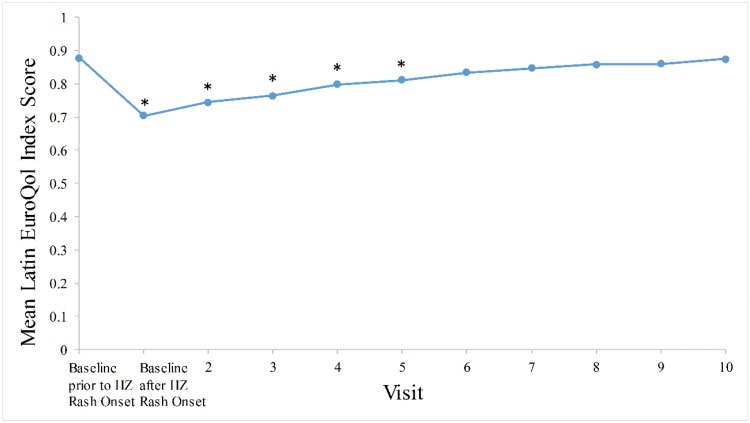
Mean Quality of Life EQ-5D Index Score over Time. *p*-values used to assess change in EQ-5D between baseline prior to HZ rash onset and baseline after HZ rash onset, as well as at days 7 to 180 from paired-samples t-tests. *Denotes statistical significance: day 0 (visit 1) (*p*<0.001); day 7 (visit 2) (*p*<0.001); day 14 (visit 3) (*p*<0.001); day 21 (visit 4) (*p*<0.001); day 30 (visit 5) (*p*=0.002); day 60 (visit 6) (*p*=0.055); day 90 (visit 7) (*p*=0.116); day 120 (visit 8) (*p*=0.217); day 150 (visit 9) (*p*=0.443); day 180 (visit 10) (*p*=0.856).

Figure 2 illustrates patients’ QoL over time as represented by EuroQoL index scores. A statistically significant (*p*<0.001) deterioration in QoL was observed prior to and immediately following rash onset, with mean (SD) EQ-5D index scores decreasing from 0.9 (0.2) to 0.7 (0.2), respectively. Furthermore, EQ-5D index scores significantly (*p*<0.05) improved between baseline and days 7 to 30. More specifically, mean (SD) index scores gradually increased from 0.7 (0.2) immediately following rash appearance to 0.7 (0.2) at day 7, 0.8 (0.2) at day 14, 0.8 (0.2) at day 21 and 0.8 (0.2) at day 30. Although QoL further improved between days 60 and 180, results were not significantly different, with baseline values prior to rash onset (*p*>0.05).

Table 2 summarizes the HZ-related HCRU and healthcare costs from rash onset to day 180. The most frequent HCRUs were visits to the ER (67.1%), followed by visits to a doctor’s office (65.8%), nursing services (30.8%), and visits to specialists (19.9%). Among patients who utilized health care resources, the highest mean number (SD) of visits were to specialists’ offices, with 5.7 (12.1) visits during the observation period, followed by the number of visits to doctors’ offices [3.5 (6.6)] and the number of times nursing services were used [3.3 (5.6)]. Among hospitalized patients (8.9%), the mean (SD) number of days spent in the hospital was 5.8 (9.0) days. The proportion of patients who purchased prescription, OTC, and alternative medications was 89.7%, 43.2%, and 2.1%, respectively. Among users of purchased medications, the average cost was 567$ BRL for prescription medications, 95$ BRL for alternative medications and 21$ BRL for OTC medications. Nearly half of patients (45.9%) needed caregiver assistance during HZ episodes, while 38.4% reported that family members missed work while taking care of patient, 8.2% of patients required an extended sick leave for a mean (SD) of 42.8 (42.9) days. In addition, 19.9% and 7.5% of patients missed a mean (SD) of 45.3 (41.3) and 13.8 (19.6) full and partial work days, respectively, due to HZ-associated disease burden.

## DISCUSSION

This study assessed the disease burden, impact on QoL, and HCRU associated with HZ in Brazil. Limited HZ epidemiological findings have been reported in Brazil. However, in a Brazilian study conducted by Castro et al., where medical records of child and adult patients were obtained from a dermatology clinic, the reported incidence rate was 5.62 cases per 1.000 person-years between 1987 and 1989 9. Additional Latin American estimates include those reported by Pérez Pérez et al. of a rate of 4.96% among maxillary surgery inpatients in a Cuban hospital in 2001 10 and by Vujacich et al. of a rate of 0.50% in Buenos Aires, Argentina between 2000 and 2005 11. In Italy, Alicino et al. reported an overall incidence of 6.42 cases per 1,000 person-years 21. In a worldwide systematic review of 63 HZ studies, Kawai et al. noted relatively similar age-specific incidences across North America, Europe and Asia. The observed variations between countries were attributed to variations in study quality and design, the assessed populations and the years the study was performed 22, though differences regarding HZ surveillance systems may have led to further variation in estimates.

To our knowledge, this is the first study to prospectively assess the disease burden related to HZ in Brazil. Overall, HZ was associated with significant impact on pain, QoL and HCRU. As reduced immunological activity may increase the risk of HZ development among individuals with latent VZV 15, resources should be provided to improve HZ prevention and management, especially as 16.5% of the Brazilian population was older than 55 years of age in 2015 23.

The study results show the considerable and rapid impact of HZ on patients’ QoL, as illustrated by the highest mean ZBPI pain scores and lowest EQ-5D QoL index scores immediately following HZ rash appearance. Although pain generally decreased over time, a negligible improvement of pain was reported during the first 14 days (days 0 to 14) following rash onset. Moreover, pain did not entirely subside at the end of the study, illustrating the long-term pain and lengthy recovery associated with HZ and PHN. The prolonged duration of pain is highlighted by the important subset of patients with PHN (49.3%), particularly as the mean (SD) pain duration of 143.1 (440.2) days was longer than the 90-day established threshold of PHN. Nonetheless, the large SD of the mean duration of pain illustrates the high degree of variability in the cohort with respect to the duration of ZAP.

The gradual decrease in pain was correlated with an improvement in QoL during the 180-day observation period. Significant changes in EuroQoL scores were observed between baseline and all visits within the first 60 days following HZ onset. However, this significance was lost from days 60 to 180 when compared to pre-HZ values, indicating a normalization of QoL over time. The results of the present study are comparable to findings of previous studies in the literature from other countries. In the UK, Gater et al. reported worst and average ZBPI scores of 5.0 and 3.9 at baseline and of 4.0 and 3.0 between days 7 and 14, respectively. The improvement in pain coincided with a concurrent amelioration of QoL 24. In Canada, Drolet et al. observed an improvement in mean [(95% confidence interval (CI)] ZBPI scores over time from 6.3 (5.9-6.7) to 3.9 (3.3-4.5) at rash appearance and 180 days later, respectively. Similarly, a slight improvement of QoL was observed in correlation with the alleviation of HZ pain as demonstrated by an increase in mean (95% CI) EuroQoL index scores from 0.59 (0.55-0.64) to 0.67 (0.61-0.73) at baseline and after 180 days, respectively 25. In a prospective study in Italy by Bricout et al., mean (SD) visual analog scale pain scores of the 89.6% of HZ patients who reported ZAP at the initial visit decreased from 5.8 (2.4) at study enrollment to 3.7 (2.3) at month 6, despite negligible changes in QoL over 6 months with respect to mean (SD) physical [39.2 (8.4) to 39.0 (9.3)] and mental [41.6 (10.4) to 39.4 (10.6)] SF-12 scores 26. The abovementioned studies further stated the inferiority of HZ patients’ QoL compared with that of the global population 24-26.

Patients reported substantial HCRU during the 180-day observation period, in agreement with the high levels of reported pain associated with HZ. As patients were recruited by the study investigator in an ER, a higher rate of ER visits as opposed to family doctor consultations (65.8%) was expected. Moreover, although only 20% of patients visited a specialist, the total number of visits were the highest observed in this study [mean (SD): 5.7 (12.1)]. This may be due to the high subset of patients with PHN under the assumption that patients with the most severe cases of HZ would require multiple consultations with specialists to alleviate their long-term symptoms. With respect to Latin America, unpublished surveys conducted by the Foundation for Infectious Diseases evaluating outpatient visits between 1993 and 2005 in Argentina reported a total of 803 HZ cases among 154,392 ambulatory consultations. Of the 302 patients with HZ between 2000 and 2005, complications arose in 20% of patients, and this rate was highest among those older than 50 years of age. Moreover, the most common complication was PHN, which was experienced by 13% of patients, followed by bacterial superinfection (5%) and ophthalmic complications (2%) 27.

Currently, HZ and PHN prophylaxis and treatment present an important unmet global need, and Brazil is no exception. While the preponderance of patients (95.9%) received HZ treatment, only 29.3% received antiviral therapy, the current standard of care in HZ management 2. This may be due to limited coverage by regional health policies of the Unified Health System 28. These assumptions are substantiated by the high utilization of antiviral therapies in studies conducted in Italy (91.5%) 26, Canada (88.9%) 13 and South Korea (52.3%-67.6%) 15,29. Additionally, although famciclovir has shown increased efficacy in reducing PHN duration 30, no patients utilized this medication in the present study. Physician or patient preference may have impacted the utilization of certain drugs. As PHN was experienced by a large subset of patients (49.3%) and is associated with a substantial impact on QoL, increased antiviral prescription, including those for famciclovir, may have led to reduced disease burden. Indeed, early antiviral treatment initiation has been shown to decrease acute HZ severity; however, published studies suggest that antiviral treatment has negligible impact on PHN incidence, though it has been shown to shorten the duration of PHN-associated pain 5.

Our study also highlights the importance of including work loss when assessing the economic value of any intervention aiming to control HZ. Among the 36 patients employed prior to HZ development, the vast majority [n=33 (91.7%)] reported any missed work days during the 180-day observation period.

The present study had several limitations. Although HZ generally affects elderly populations, the patient population in the present study, which only included patients above 50 years of age who sought HZ care, may have omitted certain HZ patients. This may have introduced a selection bias, as older HZ patients generally report worse pain and QoL scores, higher rates of analgesic use and a higher risk of complications 31,32. In addition, the utilization of health questionnaires is associated with certain limitations. Firstly, the results may have been subject to important patient variability, particularly as the main assessments were patient-reported outcomes (pain and QoL). Their use further carries the risk of recall bias, as prevalent cases were included in the study. Finally, despite the limited ability to conduct a comprehensive pharmacoeconomic analysis due to patients’ self-reporting their HZ-related costs, previous studies have shown the economic benefits of implementing a zoster vaccination program among the elderly 33. Moreover, misclassification of outcomes may have been introduced in the analysis as the diagnosis of HZ was not confirmed by laboratory testing and solely relied on physicians’ assessments of the rash and patients’ reporting of pain. Additionally, the inclusion of only one study site decreases the external validity of the results; however, the current results are in line with those reported in the literature 34.

Overall, this study highlights the significant disease burden associated with HZ and PHN. The high levels of ZAP correlated with a substantial impact on QoL over time. Although pain was generally alleviated over time, an important subset of patients experienced long-term PHN. These findings, as well as the high rates of HCRU observed in the study, support the need for the implementation of early intervention and prevention programs such as vaccination to reduce the HZ-associated disease burden in Brazil.

## AUTHOR CONTRIBUTIONS

Toniolo-Neto J, Rockett TY, Johnson KD, Monsanto HA and Acosta CJ substantially contributed to the conception of the study and interpretation of the data, with significant contributions towards critical review of the manuscript’s content. Psaradellis E and Karellis A substantially contributed to the analysis and interpretation of the data, with significant contributions towards producing the draft and critical review of the content. Rampakakis E and Sampalis JS substantially contributed to the analysis and interpretation of the data, with significant contributions towards critical review of the manuscript’s content.

### Conflicts of Interest

Joao Toniolo-Neto has consulted for Support (DANONE), Ache, MSD and Apsen and has been a speaker for MSD. Eliofotisti Psaradellis, Angela Karellis, Emmanouil Rampakakis and John S. Sampalis are employees at JSS Medical Research Inc., which is a CRO. Talita Y. Rockett has no disclosures to declare. Kelly D. Johnson is an employee at Merck & Co. Homero A. Monsanto and Camilo J. Acosta are employees and stock shareholders of Merck & Co.

## Figures and Tables

**Table 1A:** Socio-Demographic Characteristics.

Parameter		HZ Patients (N=146)
Gender, n (%)	Male	52 (35.6%)
	Female	94 (64.4%)
Age, years	Mean (SD)	69.9 (10.9)
	50-60, n (%)	30 (20.5%)
	61-70, n (%)	48 (32.9%)
	>70, n (%)	68 (46.6%)
Race, n (%)	Caucasian	115 (78.8%)
	Black	11 (7.5%)
	Asian	10 (6.8%)
	Brown	9 (6.2%)
	Mixed Race	1 (0.7%)
Highest Level of Education, n (%)	Illiterate	11 (7.5%)
	Primary/Elementary	69 (47.3%)
	Incomplete Primary	7 (4.8%)
	High School	28 (19.2%)
	Incomplete Higher Education	2 (1.4%)
	Completed Higher Education	26 (17.8%)
	Brazilian Movement of Literation (Mobral)	1 (0.7%)
	No Response	2 (1.4%)
Current Employment Status, n (%)	Full-Time	30 (20.5%)
	Part-Time	6 (4.1%)
	Unemployed	2 (1.4%)
	Temporary Leave	2 (1.4%)
	Homemaker	28 (19.2%)
	Retired	69 (47.3%)
	Other	9 (6.2%)

HZ, herpes zoster; SD, standard deviation.

**Table 1B:** Baseline Disease Characteristics.

Parameter		HZ Patients (N=146)
HZ Rash Present at Baseline, n (%)	Yes	114 (78.1%)
	No	32 (21.9%)
Primary Dermatome Region Affected, n (%)	Cervical	22 (15.1%)
	Thoracic	56 (38.4%)
	Sacral	3 (2.1%)
	Lumbar	20 (13.7%)
	Ophthalmic Branch of the Trigeminal Nerve	13 (8.9%)
	Not Applicable	32 (21.9%)
Number of Lesions in the Primary and Adjacent Dermatomes, n (%)	1-10	41 (28.1%)
	11-20	30 (20.5%)
	21-50	22 (15.1%)
	51-100	13 (8.9%)
	>100	7 (4.8%)
	Missing	1 (0.7%)
	Not Applicable	32 (21.9%)
Pain Prior to HZ Rash Onset	Patients Experiencing HZ-Associated Pain, n (%)	79 (54.1%)
	Number of Days with Pain from HZ Rash Onset^1^, mean (SD)	11.0 (23.9)
	Worst Pain Rating^1^,^2^, mean (SD)	7.4 (2.5)
	Average Pain Rating^1^,^2^, mean (SD)	5.8 (2.5)
Pain Following HZ Rash Onset	Patients Experiencing HZ-Associated Pain, n (%)	133 (91.1%)
	Number of Days with Pain from HZ Rash Onset^3^, mean (SD)	143.1 (440.2)
	Worst Pain Rating^2^,^3^, mean (SD)	8.6 (7.2)
	Average Pain Rating^2^,^3^, mean (SD)	5.5 (2.5)
Post-Herpetic Neuralgia^4^, n (%)	Yes	72 (49.3%)
	No	74 (50.7%)
HZ Treatment^6^, n (%)	Any HZ Treatment	140 (95.9%)
	Analgesics^5^	50 (34.2%)
	Metamizole	19 (13.0%)
	Acetaminophen	17 (11.6%)
	Adiphenine hydrochloride/Dipyrone/Promethazine hydrochloride	10 (6.8%)
	Paracetamol	6 (4.1%)
	AAB	5 (3.4%)
	Hyoscine Butyl bromide	2 (1.4%)
	Aspirin	1 (0.7%)
	Caffeine/Metamizole/Orphenadrine Citrate	1 (0.7%)
	Antivirals	40 (27.4%)
	Acyclovir	33 (22.6%)
	Valacyclovir	8 (5.5%)
	Topicals	31 (21.2%)
	Anticonvulsants	13 (8.9%)
	Antidepressants	13 (8.9%)
	Opioids	12 (8.2%)
	Steroids	9 (6.2%)
	Anti-inflammatories	5 (3.4%)
	Anti-hypertensives	5 (3.4%)
	Vitamins	5 (3.4%)
	Anxiolytics/Benzodiazepines	3 (2.1%)
	Antibiotics	0 (0.0%)
	Other^7^	65 (44.5%)

AAB, alcohol extract of *Achyranthes bidentata*; HZ, herpes zoster; SD, standard deviation.

1 Based on available data: n=63.

2 Measured with the Initial Zoster Impact Questionnaire. Pain score scale: 0, no pain; 10, worst pain.

3 Based on available data: n=133.

4 Defined as ZBPI pain scores of at least 3 at least 90 days following rash onset.

5 Patients may have reported the use of more than one analgesic.

6 Each patient was counted once per medication type.

7 Including antacids, hormones, antihistamines, anti-hyperuricemics, chemotherapy, anticoagulants, anti-arrhythmia medications, asthma medications, diuretics and hypoglycemic agents.

**Table 2:** Health Care Utilization, Patient-Reported Expenses, and Work-Related Impacts.

	Total Number of Patients (N=146)	
**Type of HCRU**	**n (%)**^1^	**Mean (SD)**^2^
Visit to Doctor’s Office	96 (65.8%)	3.5 (6.6) visits^3^
Visit to Emergency Room	98 (67.1%)	3.1 (3.0) visits^3^
Visit to Specialist	29 (19.9%)	5.7 (12.1) visits^3^
Visit to Psychiatrist/Psychologist/Counselor	2 (1.4%)	4.0 (NC) visits^3^
Ambulance Service	4 (2.7%)	1.0 (NC) times^3^
Nursing Service	45 (30.8%)	3.3 (5.6) times^3^
Hospitalization	13 (8.9%)	5.8 (9.0) days^4^
**Type of Health Care Cost**		
Out-of-Pocket Expenses*	**n (%)**^1^	**Mean (Range)**^2^
Purchased Prescription Medications for HZ	131 (89.7%)	567.0 (10.0-6595.0) BRL $^5^
Purchased Over-The-Counter Medications for HZ	63 (43.2%)	20.6 (5.0-79.0) BRL $^5^
Purchased Alternative Medications for HZ	3 (2.1%)	94.5 (39.0-150.0) BRL $^5^
Patient Needed Help of Others for Care During HZ Episodes	67 (45.9%)	NA
Hired Caregiver/Increased Caregiver Use during HZ Episodes	4 (2.7%)	712.5 (50-1800) BRL $^5^
**Work and Productivity**	**n (%)**^1^	**Mean (SD)**^2^
Family Member Missed Work while Taking Care of Patient with HZ	56 (38.4%)	8.0 (28.2) days^4^
Employed Prior to HZ Episodes	36 (24.7%)^6^	NA
Missed an Entire Work Day due to HZ	29 (19.9%)	45.3 (41.3) times^7^
Missed a Part of a Work Day due to HZ	11 (7.5%)	13.8 (19.6) times^7^
Went on Extended Sick Leave due to HZ Episodes	12 (8.2%)	42.8 (42.9) days^4^

* Cost estimates pertain to year 2009.

1 Represents the proportion of total population.

2 Results are based on patients with available data among patients who used respective health care resource.

3 Represents the number of times each resource was used.

4 Represents the duration in days.

5 Represents the mean cost of each resource use per patient in Brazilian Real (BRL).

6 Of the 36 patients employed prior to HZ development, 33 reported any missed days of work during the 180-day period (including entire work days, partial work days or extended sick leave).

7 Represents the number of times work was missed.

HCRU, health care resource utilization; NA, not applicable; NC, non-calculable; SD, standard deviation.
